# Preclinical molecular imaging: development of instrumentation for translational research with small laboratory animals

**DOI:** 10.1590/S1679-45082016AO3696

**Published:** 2016

**Authors:** Jorge Mejia, Ana Claudia Camargo Miranda, Ana Claudia Ranucci Durante, Larissa Rolim de Oliveira, Marycel Rosa Felisa Figols de Barboza, Katerin Taboada Rosell, Daniele Pereira Jardim, Alexandre Holthausen Campos, Marilia Alves dos Reis, Marcela Forli Catanoso, Orfa Yineth Galvis-Alonso, Francisco Romero Cabral

**Affiliations:** 1 Hospital Israelita Albert Einstein, São Paulo, SP, Brazil.; 2 Universidade Federal de São Paulo, São Paulo, SP, Brazil.; 3 Faculdade de Medicina de São José do Rio Preto, São José do Rio Preto, SP, Brazil.

**Keywords:** Models, animal, Tomography, emission-computed, single-photon, Likelihood functions

## Abstract

**Objective:**

To present the result of upgrading a clinical gamma-camera to be used to obtain *in vivo* tomographic images of small animal organs, and its application to register cardiac, renal and neurological images.

**Methods:**

An updated version of the miniSPECT upgrading device was built, which is composed of mechanical, electronic and software subsystems. The device was attached to a Discovery VH (General Electric Healthcare) gamma-camera, which was retired from the clinical service and installed at the *Centro de Imagem Pré-Clínica* of the *Hospital Israelita Albert Einstein*. The combined system was characterized, determining operational parameters, such as spatial resolution, magnification, maximum acceptable target size, number of projections, and acquisition and reconstruction times.

**Results:**

Images were obtained with 0.5mm spatial resolution, with acquisition and reconstruction times between 30 and 45 minutes, using iterative reconstruction with 10 to 20 iterations and 4 projection subsets. The system was validated acquiring *in vivo* tomographic images of the heart, kidneys and brain of normal animals (mice and adult rats), using the radiopharmaceuticals technetium-labeled hexakis-2-methoxy-isobutyl isonitrile (^99m^Tc-Sestamibi), technetium-labeled dimercaptosuccinic acid (^99m^Tc-DMSA) and technetium-labeled hexamethyl propyleneamine oxime (^99m^Tc-HMPAO).

**Conclusion:**

This kind of application, which consists in the adaptation for an alternative objective of already existing instrumentation, resulted in a low-cost infrastructure option, allowing to carry out large scale *in vivo* studies with enhanced quality in several areas, such as neurology, nephrology, cardiology, among others.

## INTRODUCTION

Molecular Imaging is an area of Medicine that encompasses a group of imaging techniques that allows visualize, characterize and quantify, non-invasively, biological processes and phenomena that occur at molecular and celular levels, inside living organisms.^(1)^ These images may be two dimensional or volumetric, static or dynamic and may use different energy bands. In this group of techniques, we can include the single photon emission computed tomography (SPECT), positron emission tomography (PET), magnetic resonance imaging (MRI), computerized tomography, ultrasound, fluorescence imaging, and bioluminescence.^(2)^ These tools are considered standard in the clinical environment,^(3)^ with the exception of fluorescence and bioluminescence, which use infrared or visible radiation, and were developed in the preclinical environment.^(4)^

Recently, a great effort has been done to transfer SPECT and PET techniques to the preclinical environment,^(5–10)^ where rats and mice are the most frequently used animals to study and treat models of human diseases. Specifically, SPECT is a technique to obtain images in which a drug linked to a gamma ray emitting element, with affinity for an organ or process of interest, is injected into the patient. Images of the target, called projections, are taken from different viewpoints. Using the appropriate computerized tools, these projections are combined to generate a tomographic image of the target. Differently from invasive techniques to evaluate experimental protocol effects, in which euthanasia and dissection of the target organ or tissue are normally used, molecular imaging techniques allow us to visualize the evolution of the subject of the study with minimal interference. Thus, the animal can be used as its own control and be repeatedly assessed at different time points along the experiment, which reduces the effects of differences between the animals, improving the statistical quality of the collected data. Moreover, since an experimental group for each evaluation moment is not necessary, there is a significant reduction in execution costs, as well as a better accordance with the ethical considerations regarding animal use in experimentation.^(11)^

However, considering the size of these animals, making images in this environment faces challenges associated with spatial resolution and sensitivity. To solve this issue, different research groups work to develop strategies to adapt existing clinical equipments,^(10,12,13)^ or to build specific devices for laboratory animals,^(14–17)^ each one favoring different aspects, such as cost, reuse of deactivated equipment, use during spare time and equipment availability. In any case, developing the necessary instrumentation to produce these images, allows us to understand the involved parameters and control the associated sources of errors, thus obtaining the appropriate results, with an investment that is adequate to the formulated question.

Bearing this in mind, the *Hospital Israelita Albert Einstein* has transferred a SPECT gamma-camera from the clinical service to the *Centro de Imagem Pré-Clínica*, located in the Experimental and Surgical Training Center (CETEC), where it was installed and adapted to obtain images of small animals. The adaptation device corresponds to an update of the previously-developed miniSPECT device, which was fitted to the specific camera.^(13)^

## OBJECTIVE

To present the result of the updating of a clinical gamma-camera, to use it as a dedicated instrument to obtain tomographic images of the organs of small laboratory animals, and its application to obtain cardiac, renal and neurological images.

## METHODS

### Description of the adapted equipment

We used a SPECT gamma-camera that was removed from the clinical service, model Discovery VH (General Electric Healthcare, Milwaukee, WI, USA), containing two NaI(Tl) detectors, with a useful area of 540mm×400mm and 9.5mm thick, intrinsic spatial resolution of 3.9mm and energy resolution of 10% at 140keV. The way it was installed, the proprietary software allows records in static, dynamic or SPECT modes. In the static mode, individual images are recorded, with integration time determined by the user. In the dynamic mode, the system records a sequence of images with a previously selected integration time, without detector movement. In the SPECT mode, the detectors move, allowing record a set of images from different angles around the target. The images can be registered in matrices of up to 1,024×1,024 pixels and it is possible to synchronize image records to the electrocardiogram (ECG). In this work, the camera was used in the dynamic mode, and the animal was rotated to record individual projections.

**Figure 1:**
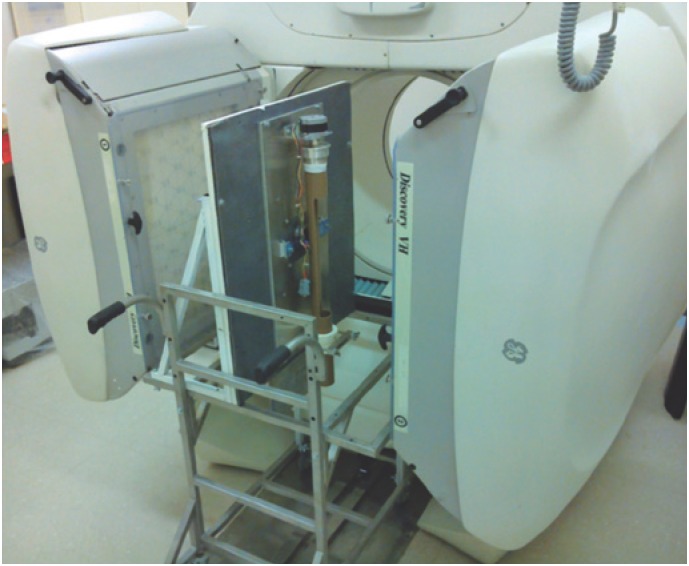
Image of the gamma-camara Discovery VH and the miniSPECT upgrading device

The adaptation device, which we called miniSPECT, consists of three subsystems: mechanical, electronic and software.^(13)^ The mechanical subsystem includes a set of plastic tubes, transparent to gamma radiation, for the positioning of different size targets, which are rotated by a stepper motor. There are also pinhole collimators made on lead slabs, which project the images over the detector. We tested collimators that consist of small tungsten inserts, mounted on a lead slab or in a pinhole directly perforated on the lead block. The collimators are shaped as a double cone, whose vertices coincide in the center of the piece of which the collimator is made. The collimators are mounted over an aluminum base plate covered with lead, which serves as shield, to avoid unwanted radiation incidence on the detector. On this plate, target-holder tubes and the target rotation system are fixed. Finally, the base plate is mounted over a cart that allows the device to be positioned in front of the detector, regardless of the camera used. The developed device can be seen in figure 1.

The electronic subsystem consists of a microcontrolled system, which rotates the target-holder tube in front of the collimator, synchronized with the camera's image recording procedure. This subsystem allows the selection of the number of projections and time per projection within a group of pre-programmed options, and controls the modes “on” and “stand by”. Finally, the software subsystem consists of an iterative tool for three-dimensional reconstruction, which combines the recorded projections with a physical model of the instrument using the maximum likelihood algorithm^(18)^ to generate the target image that better corresponds to the recorded images. This tool was developed in C language (Microsoft Visual Studio C/C++). Visualization and analysis of the final images were performed using free software tools, such as ImageJ (http://rsbweb.nih.gov/ij/) and Amide (http://amide.sourceforge.net). As an alternative, it is possible to use the Ordered Subset Expectation Maximization (OSEM) technique. With it, subgroups of projections were used to make intermediate reconstructions, which served as input models for the subsequent iterations. This way, it was possible to significantly reduce the processing time, accelerating the image reconstruction process.^(19)^

### Determination of the system spatial resolution: phantoms

In SPECT tomography instruments that use pinhole collimators, spatial resolution is a function of these factors: detector's intrinsic resolution, magnification factor, pinhole diameter, collimator material, and emission energy of the radionuclide. The first factor determines the size with which the gamma-camera records the image of a small source projected directly on the detector, depends on the camera model and, in this project, remains invariable. The magnification factor is given by the ratio between the collimator-detector and collimator-target distances. The shorter the collimator-target distance, or the longer the collimator-detector distance, the greater the image magnification, which increases the separation between the images generated by two neighboring points in the target. Magnification is limited by the target size and by the detector's useful area, making it necessary for the target, during its rotation, to always be projected in its totality onto the detector's useful area. Finally, the pinhole diameter affects the projection size of a small source, in such a way that, the smaller the orifice, the smaller the area occupied by the projection of this source, improving the ability to separate images from two near points in the target. Additionally, the spatial resolution depends on the collimator material and the energy of the radionuclide used, due to photon penetration through pinhole borders, in such a way that, for greater energy or less dense materials, the orifice is effectively larger than the physical diameter.^(20)^ In this work, we were limited to an energy of 140keV, which corresponds to the emission by ^99m^Tc, and we tested lead and tungsten collimators.

To determine the instrument's spatial resolution, we used two different phantoms: (a) a hot-rod phantom or Jaszczak phantom, and (b) a group of parallel capillaries. The first phantom consisted of an acrylic piece with four sets of orifices of 0.5, 1.0, 1.5 and 2.0mm of diameter, with a distance between orifice borders equal to the diameter of the orifices of that set. This piece was placed in a plastic tube filled with sodium pertechnetate (Na-^99m^TcO_4_^−^), sealed to avoid leaks. The second phantom consisted of a group of six parallel capillaries mounted into a triangular shape, filled with Na-^99m^TcO_4_^−^. The distance between the borders of the filled spaces of contiguous capillaries was of 0.5mm. Both phantoms were filled with a concentration of 1mCi/mL. Each phantom was positioned in the imaging device and two sets of images were made: one with the Jaszczak phantom, with a magnification factor of 7x and a 1.5mm lead pinhole collimator, and the second set with the capillary phantom, with a magnification factor of 9x and a 0.5mm tungsten pinhole collimator. In both cases, 40 projections were recorded, of 60 seconds each. The projections were processed to generate the three-dimensional image of the Na-^99m^TcO_4_^−^ distribution in the phantom using the iterative software tool, with 20 iterations, 4 projection subgroups and smoothing with a 1.5-pixel wide Gaussian kernel every two iterations.

### Images of normal animal organs Animals

We used two male Swiss mice, normal, with a weight around 30g and one male Wistar rat, normal, weighing 350g. The animals were kept at CETEC's experimentation vivarium, with a light-dark cycle of 12 hours, room temperature controlled at 22±2°C and with free access to water and food. For image recording, radiopharmaceuticals were administered intravenously through the tail vein. After that, the animals were anaesthetised with ketamine:xylazine (75:10mg/kg), respecting the time between the injection and beginning of records. After image acquisition, the animals were kept in a shielded room in the vivarium for 48 hours until total decay of the radionuclide. Afterwards, they were transferred to the conventional experimentation vivarium. All procedures were carried out in accordance with the Brazilian Guidelines on Care and Use of Animals for Scientific Research or Teaching Activities (DBCA) and were approved by the Ethics Committee for the Use of Animal Experimentation from *Hospital Israelita Albert Einstein*, protocol 2.359/15.

### Kidneys – DMSA-^99m^Tc

Dimercaptosuccinic acid labeled with ^99m^Tc (DMSA^99m^Tc) is used in nuclear medicine to study kidney function and visualise abnormalities in the parenchyma, taking advantage of the fact that it is removed from the plasma by renal proximal tubular cells and accumulates in the cortex, with low urine elimination.^(21,22)^ This radiopharmaceutical is highly bound to plasma proteins and is excreted by glomerular filtration and tubular secretion. A total of 130MBq of DMSA-^99m^Tc were administered in a conscious mouse. The animal was anaesthetized two hours after the injection and positioned horizontally in the target-holder tube. A total of 40 projections of 30 seconds each were recorded, using a 1.5mm collimator with a 7x magnification. Projections were processed to produce the tomographic image of the target organ, using 10 iterations and 4 projection subgroups.

### Heart – Sestamibi-^99m^Tc

Hexakis-2-methoxy-isobutyl isonitrile labeled with ^99m^Tc (Sestamibi-^99m^Tc) is a radiopharmaceutical used to study myocardial perfusion, which accumulates in the myocytes proportionally to the blood flow.^(23)^ This radiopharmaceutical is not highly bound to plasma proteins, with quick blood clearance, and is excreted through the renal and hepatobiliary systems and through the intestines. A total of 186MBq of Sestamibi-^99m^Tc were administered to a conscious mouse. The animal was anaesthetized two hours after the injection and positioned vertically in the target-holder tube. A total of 40 projections of 30 seconds each were recorded, using a 1.5mm collimator with a 9x magnification. To produce tomographic images of the target organ, 10 iterations and 4 projection subgroups were used.

### Brain – HMPAO-^99m^Tc

Hexamethyl-propyleneamine oxime labeled with ^99m^Tc (HMPAO-^99m^Tc) and ethylene cysteine diethyl ester dihydrochloride labeled with ^99m^Tc (ECD-^99m^Tc) are radiopharmaceuticals used in clinical practice for cerebral perfusion studies. They are lipophilic agents, which ensures their passage through the blood brain barrier, that accumulate in the intracellular environment proportionally to the blood flow.^(23)^ Differently from HMPAO-^99m^Tc, ECD-^99m^Tc is not retained in the brains of rats or rabbits,^(24,25)^ which justifies our choice for HMPAO-^99m^Tc in this experiment. Its excretion occurs through the hepatobiliary and renal systems. Thus, 370MBq of HMPAO-^99m^Tc were administered to an anaesthetized rat. Immediately after, the animal was positioned vertically in the target-holder tube. A total of 40 projections of 30 seconds each were recorded, using a 1.5mm collimator with a 5.5x magnification. The tomographic image of the target organ was produced using 20 iterations and 4 subgroups of projections.

## RESULTS

### Spatial resolution

**Figure 2:**
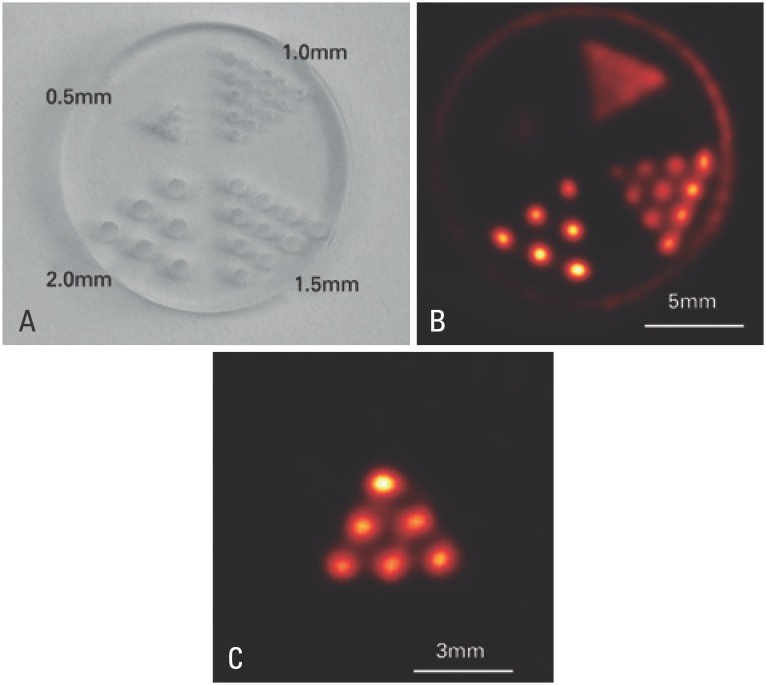
Spatial resolution. (A) Image of the Jaszczak phantom indicating the orifice sizes and the space between them. (B) Transaxial section of the radiotracer distribution image in the Jaszczak phantom, in which the 1.5mm bars can be easily identified. (C) Transaxial section of the radiotracer distribution image in the capillary phantom in which the contiguous capillaries are individually identified

In figure 2, we can observe a picture of the Jaszczak phantom and transaxial sections through the images of the Na-^99m^TcO_4_^−^ distribution in both phantoms. In the first case, which corresponds to a configuration for the study of targets the size of young rats, it was possible to see that a spatial resolution between 1.0 and 1.5mm was obtained. In the second case, which corresponds to a configuration for the study of targets the size of mice, contiguous capillaries were identified individually, corresponding to a spatial resolution better than 0.5mm.

### Renal image

**Figure 3:**
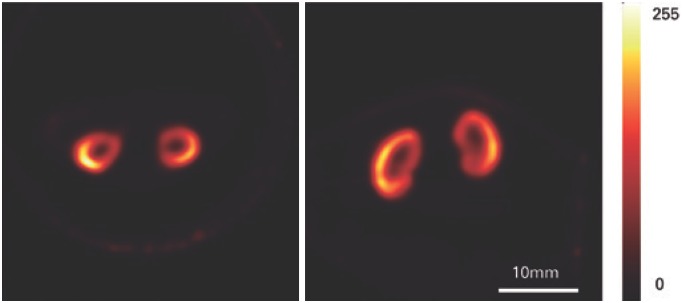
Renal image with DMSA-^99m^Tc. Horizontal and transversal sections through the reconstructed model of the radiopharmaceutical DMSA-^99m^Tc distribution in the kidneys of the normal mouse

In figure 3, we illustrate the results of the renal function imaging of a normal mouse. At the left, we observe a transversal section of the tomographic image of the kidneys, and, at the right, a horizontal section of the same image. We can see that the kidneys had dimensions of 3.5mm in diameter and 6.5mm in height. Moreover, integrity of the renal parenchyma can be verified.

### Cardiac image

**Figure 4:**
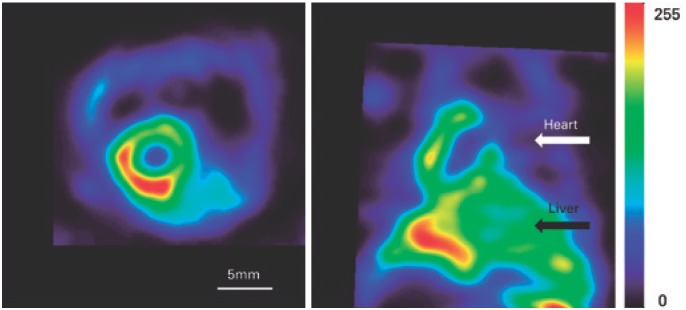
Cardiac image with Sestamibi-^99m^Tc. Horizontal and transversal sections through the reconstructed model of the radiopharmaceutical Sestamibi-^99m^Tc distribution in the heart of a normal mouse

In figure 4, the result of the cardiac perfusion imaging of a normal mouse is shown. At the left, a transversal section of the tomographic image, and at the right, a horizontal section of the same image. Due to the anatomical proximity of the heart and the liver, associated to the hepatic excretion of Sestamibi, hepatic activity overlapped with the inferior left ventricular wall, even if the animal is kept in the vertical position.

### Cerebral perfusion image

**Figure 5:**
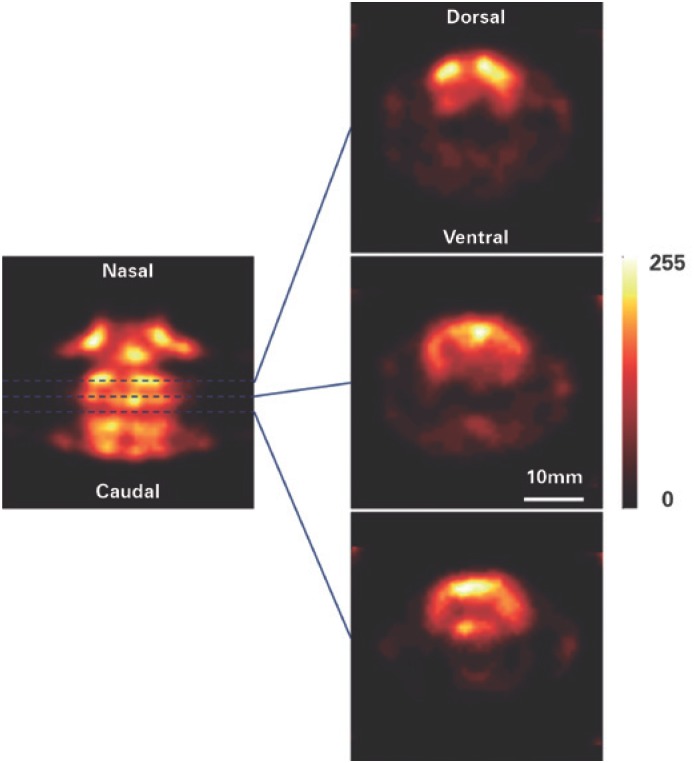
Cerebral perfusion image with HMPAO-^99m^Tc. Horizontal section and three coronal sections through the reconstructed model of the radiopharmaceutical HMPAO-^99m^Tc distribution in the brain of a normal rat

In figure 5, we present the results of a cerebral perfusion image of a young rat. At the left, a horizontal section through the tomographic image, and at the right, three transversal sections of the same image at different heights.

## DISCUSSION

Recently, the need to apply molecular imaging techniques in the preclinical environment has been widely recognized. Most of these techniques were developed for clinical use, where organs and structures of interest have dimensions of several centimeters. In the preclinical environment, however, where experiments are done in small animals, target structures are only a few millimetres in size, or sometimes smaller. Thus, molecular imaging techniques are faced with important challenges associated to spatial resolution and sensitivity.

To overcome these challenges, two approaches have been followed: adapting clinical instruments, or developing devices that are specific for small animals. In the former approach, equipment developed to evaluate human organs, with relatively well-known technology, are modified by the installation of electromechanical devices to get closer to the requirements of spatial resolution and sensitivity for the new application.^(12,13,26–28)^ This approach has the advantage that costs are significantly lower once the detection device is already installed and can be used during spare times, or it is an older machine which has been deactivated from clinical use, even though it remains fully functional. In the latter approach, detectors, collimators and image reconstruction tools are developed and built to be used specifically in preclinical environments, with targets ranging from mice to adult rats.^(15,16,29,30)^

Regardless of the selected approach, the use of molecular imaging techniques allows evaluate experimental protocol evolution at intermediate stages, keeping the animal alive, which significantly reduces the number of experimental groups and, with that, costs and execution time, and also leads to a better quality of results.

In this application, we tested the use of an updated version of the adaptation device miniSPECT in a clinical gamma-camera Discovery VH from General Electric Healthcare, and we verified that it is possible to obtain spatial resolution better than 0.5mm, using a single pinhole collimator of 0.5mm in diameter and a magnification of 9x, which corresponds to an appropriate configuration for images of mouse organs. In a configuration appropriate for rat organs, we reached a spatial resolution better than 1.5mm. Images of different mouse and rat organs were obtained, in all cases, with a recording time under 30 minutes. Recorded projections were processed and the three-dimensional images of the radiopharmaceutical distribution were produced using a computational tool based on the maximum likelihood algorithm. This tool was adapted for the specific conditions of this application, from a previous version implemented in C Language, using the free version of the Visual Studio C++ compiler.^(13)^ Images of myocardial perfusion, renal functionality, and cerebral perfusion of normal animals were obtained, showing the technique applicability in these areas. Considering that the presented solution is based on the use of an equipment removed from the nuclear medicine service, that the mechanical and electronic subsystems use components that are easy to acquire in the local market, and that the software subsystem was developed in our laboratory, the combined equipment implementation cost is significantly reduced, in comparison to commercial equipments specific for experimentation animals.

In the future, a second device should be built, allowing us to simultaneously use both detectors, which will give us a reduction in recording time or in the activity of the radiopharmaceutical administered to the animal. We will work on the use of multipinhole collimators, which should allow us to make more efficient use of detectors and to reduce recording time. Finally, we will include a tool for synchronized records with ECG, which should improve image quality and facilitate specific cardiology studies.

## CONCLUSION

We were able to develop low-cost instrumentation, benefiting from the reuse of equipment retired from its original function, reaching results that were comparable to those produced by commercial equipment specifically produced for this application. In addition, considering that the apparatus is installed in *Centro de Imagem Pré-Clínica*, at the Experimental and Surgical Training Center, it will be possible to carry out this type of experiment in a continuous way, becoming a permanently available tool for research in different areas. Finally, there is still room for improvement in the equipment, which will allow us to conduct enhanced studies in specific areas.
